# Nipocalimab in the management of generalized myasthenia gravis: a new targeted therapeutic approach

**DOI:** 10.1097/MS9.0000000000004390

**Published:** 2025-11-24

**Authors:** Muddassir Khalid, Ehtisham Haider, Afzaal Khan, Noor-Ul-Eman Haider

**Affiliations:** a Department of Medicine, Nishtar Medical University, Multan, Pakistan; b Department of Medicine, Punjab Medical College, Faisalabad, Pakistan; c Department of Medicine, Al Nafees Medical College, Islamabad, Pakistan; d Department of Medicine, Khyber Medical College, Peshawar, Pakistan

*Dear Editor,*

Myasthenia gravis is a chronic autoimmune, antibody-mediated neuromuscular disorder characterized by weakness of skeletal muscle. It often involves bulbar, limb, neck, and respiratory muscles, along with ocular manifestations, due to pathogenic autoantibodies against the acetylcholine receptor (anti-AChR) or muscle-specific kinase (anti-MuSK). These antibodies impair synaptic signaling at the neuromuscular junction, resulting in a spectrum of clinical features ranging from mild to life-threatening^[1]^. Conventional therapies include acetylcholinesterase inhibitors, corticosteroids, and broad-spectrum immunosuppressants. While these agents provide clinical benefit, they are associated with significant adverse effects: bradycardia, hypotension, paradoxical muscle weakness, osteoporosis, diabetes, bone marrow suppression, and hepatotoxicity. In addition, many patients remain dependent on invasive procedures, such as plasmapheresis, which cannot be maintained in the long term^[2]^. Generalized myasthenia gravis (gMG) therefore remains a therapeutic dilemma, with most patients requiring chronic immunosuppression that carries cumulative toxicity^[3]^.

Targeted treatments such as complement inhibitors have shown benefit in subsets of patients but fail to adequately address the heterogeneity of antibody subtypes encountered in the clinic^[4]^. This creates a pressing need for therapeutic strategies that are effective across antibody subtypes, safer, and more sustainable. Nipocalimab (Imaavy), a novel neonatal Fc receptor (FcRn) blocker, represents a promising approach. By binding to FcRn, it disrupts IgG recycling and accelerates degradation of all IgG subclasses, including pathogenic anti-AChR and anti-MuSK antibodies^[5]^. This reduction in circulating autoantibodies restores neuromuscular transmission and improves clinical outcomes. Importantly, IgM, IgA, and cell-mediated immunity are spared, minimizing the risk of infections and generalized immunosuppression. Unlike corticosteroids or conventional immunosuppressants, nipocalimab does not cause osteoporosis, diabetes, hypertension, or direct hepatic or renal toxicity^[3,5]^. Clinical evidence supports these mechanistic advantages. In early trials, patients receiving nipocalimab demonstrated rapid improvement in muscle strength and quality-of-life measures, with onset of benefit observed within weeks. The decline in circulating antibody levels closely paralleled clinical improvement, supporting the causal mechanism^[6]^. Moreover, nipocalimab was safe and well-tolerated, with fewer adverse events compared to conventional immunosuppressants^[6,7]^.

Despite these advantages, several limitations deserve consideration. Prolonged IgG suppression raises concerns about hypogammaglobulinemia and increased risk of infection, although current data suggest minimal increases in serious infections^[7]^. Long-term safety data are still limited, particularly regarding the risk of relapse after discontinuation and the effects on vaccination efficacy. Accessibility is another challenge: biologic therapies carry substantial financial costs, and availability may be restricted in resource-limited settings. Finally, real-world effectiveness and head-to-head comparisons with other targeted therapies, including complement inhibitors and emerging FcRn blockers, remain to be determined^[4,7]^.

In conclusion, nipocalimab offers a targeted, steroid-sparing therapeutic option that can potentially transform the management of gMG. By reducing pathogenic IgG autoantibodies while preserving other immune functions, it addresses the heterogeneity of autoantibody subtypes and minimizes the toxicities of conventional therapies. Future research should focus on long-term outcomes, optimal treatment duration, cost-effectiveness, and integration into individualized therapeutic algorithms. With continued clinical validation, nipocalimab may reduce dependence on steroids and invasive therapies, significantly improving patient outcomes in gMG^[5–7]^.

This letter to the editor complies with the TITAN guideline^[8]^.

## Data Availability

Not applicable.
